# Aqua­(3-hydroxy­benzoato-κ*O*)bis­(1,10-phenanthroline-κ^2^ *N*,*N*′)cobalt(II) 3-hydroxy­benzoate penta­hydrate

**DOI:** 10.1107/S1600536808001815

**Published:** 2008-01-23

**Authors:** Jun-Hua Li, Jing-Jing Nie, Jian-Rong Su, Duan-Jun Xu

**Affiliations:** a Department of Chemistry, Zhejiang University, People’s Republic of China

## Abstract

The crystal structure of the title compound, [Co(C_7_H_5_O_3_)(C_12_H_8_N_2_)_2_(H_2_O)](C_7_H_5_O_3_)·5H_2_O, consists of Co^II^ complex cations, uncoordinated hydroxy­benzoate anions and uncoord­inated water mol­ecules. The Co^II^ ion is coordinated by two phenanthroline ligands, a water mol­ecule and a 3-hydroxy­benzoate anion, and displays a distorted octa­hedral geometry. π–π stacking is observed between parallel phenanthroline ligands, the face-to-face separations being 3.454 (19) and 3.435 (7) Å. An extensive hydrogen-bonding network helps to stabilize the crystal structure. The hydroxybenzoate ligand is disordered over two positions, with site occupancy factors 0.6 and 0.4. One solvent water molecule is also disordered over two positions, with site occupancy factors 0.6 and 0.4.

## Related literature

For general background, see: Hu *et al.* (2002 ▶); Li *et al.* (2005 ▶). For related structures, see: Su *et al.* (2005 ▶); Pan *et al.* (2006 ▶).
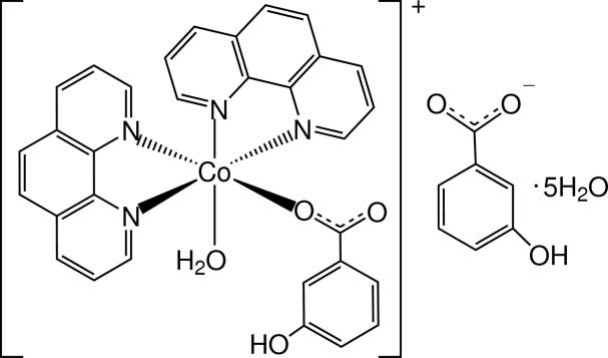


## Experimental

### 

#### Crystal data

[Co(C_7_H_5_O_3_)(C_12_H_8_N_2_)_2_(H_2_O)](C_7_H_5_O_3_)·5H_2_O
*M* *_r_* = 801.65
Triclinic, 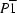

*a* = 12.3404 (12) Å
*b* = 12.6844 (16) Å
*c* = 13.561 (2) Å
α = 101.507 (5)°
β = 101.781 (4)°
γ = 111.823 (7)°
*V* = 1838.4 (4) Å^3^
*Z* = 2
Mo *K*α radiation
μ = 0.54 mm^−1^
*T* = 295 (2) K
0.32 × 0.28 × 0.20 mm

#### Data collection

Rigaku R-AXIS RAPID IP diffractometer
Absorption correction: multi-scan (*ABSCOR*; Higashi, 1995 ▶) *T*_min_ = 0.850, *T*_max_ = 0.905
11865 measured reflections
6450 independent reflections
4935 reflections with *I* > 2σ(*I*)
*R*_int_ = 0.050

#### Refinement

*R*[*F* ^2^ > 2σ(*F* ^2^)] = 0.070
*wR*(*F* ^2^) = 0.247
*S* = 1.02
6450 reflections
577 parameters
4 restraints
H-atom parameters constrained
Δρ_max_ = 0.42 e Å^−3^
Δρ_min_ = −0.69 e Å^−3^

### 

Data collection: *PROCESS-AUTO* (Rigaku, 1998 ▶); cell refinement: *PROCESS-AUTO*; data reduction: *CrystalStructure* (Rigaku/MSC, 2002 ▶); program(s) used to solve structure: *SIR92* (Altomare *et al.*, 1993 ▶); program(s) used to refine structure: *SHELXL97* (Sheldrick, 2008 ▶); molecular graphics: *ORTEP-3 for Windows* (Farrugia, 1997 ▶); software used to prepare material for publication: *WinGX* (Farrugia, 1999 ▶).

## Supplementary Material

Crystal structure: contains datablocks I, global. DOI: 10.1107/S1600536808001815/ng2420sup1.cif

Structure factors: contains datablocks I. DOI: 10.1107/S1600536808001815/ng2420Isup2.hkl

Additional supplementary materials:  crystallographic information; 3D view; checkCIF report

## Figures and Tables

**Table 1:** Selected bond lengths (Å)

Co—O1	2.107 (3)
Co—O1*A*	2.116 (14)
Co—O1*B*	2.05 (2)
Co—N1	2.154 (4)
Co—N2	2.153 (3)
Co—N3	2.155 (4)
Co—N4	2.185 (3)

**Table 2:** Hydrogen-bond geometry (Å, °)

*D*—H⋯*A*	*D*—H	H⋯*A*	*D*⋯*A*	*D*—H⋯*A*
O1—H1*A*⋯O11	0.93	1.77	2.671 (5)	162
O1—H1*B*⋯O2*A*	0.88	1.95	2.78 (2)	159
O3*A*—H1⋯O1*W*^i^	0.88	1.78	2.650 (10)	170
O13—H13⋯O2*A*^ii^	0.97	1.86	2.81 (2)	166
O1*W*—H1*AW*⋯O12	0.97	1.85	2.810 (7)	168
O1*W*—H1*BW*⋯O1*W*^iii^	0.89	2.33	2.833 (8)	116
O2*W*—H2*AW*⋯O11	0.88	1.99	2.851 (11)	167
O2*W*—H2*BW*⋯O2*A*	0.89	2.04	2.90 (2)	162
O3*W*—H3*AW*⋯O12	0.91	1.92	2.824 (10)	168
O3*W*—H3*BW*⋯O5*WA*^iv^	0.90	1.94	2.589 (16)	127
O3*W*—H3*BW*⋯O5*WB*	0.90	1.98	2.87 (3)	168
O4*W*—H4*AW*⋯O5*WA*^iv^	0.93	2.05	2.66 (2)	122
O4*W*—H4*AW*⋯O3*W*	0.93	2.39	3.284 (17)	161
O4*W*—H4*BW*⋯O2*W*	0.89	1.82	2.569 (18)	140
O5*WA*—H5*A*1⋯O13^v^	0.88	2.13	2.851 (13)	138
O5*WA*—H5*A*2⋯O3*A*	0.84	1.91	2.754 (14)	176
O5*WB*—H5*B*2⋯O1*W*^iii^	0.89	2.16	2.955 (17)	148

Symmetry codes: (i) 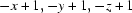; (ii) 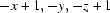; (iii) 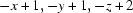; (iv) 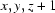; (v) 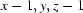.

**Fig. 1.**
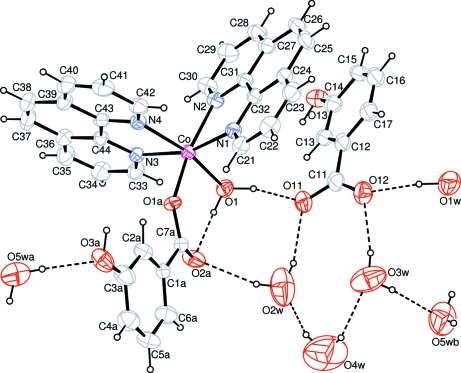
The molecular structure of the title compound with 30% probability displacement (arbitrary spheres for H atoms). One of disordered components has been omitted for clarity.

**Fig. 2.**
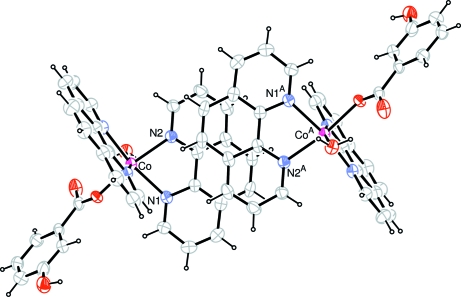
A diagram showing π-π stacking between N1-containing phen rings [symmetry code: (A) 1 - x,-y,-z].

**Fig. 3.**
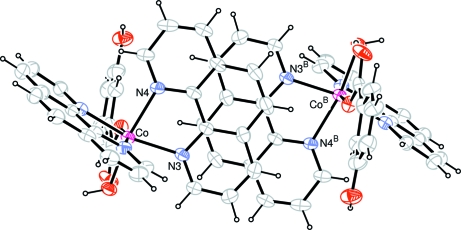
A diagram showing π-π stacking between N3-containing phen rings [symmetry code: (B) 2 - x,1 - y,1 - z].

## supplementary crystallographic information

### Comment

As part of our ongoing investigation on the nature of aromatic stacking in crystals of metal complexes (Hu *et al.*, 2002; Li *et al.*, 2005), the title compound has been prepared and its crystal structure is presented here.

The crystal consists of Co^II^ complex cations, uncoordinated 3-hydroxybenzoate (HBA) anions and lattice water molecules. The Co^II^ ion is coordinated by two 1,10-phenanthroline (phen), one water molecule and one HBA anion in a distorted octahedral geometry (Fig. 1 and Table 1). This is similar to that found in aqua(4-hydroxybenzoato)bis(phenanthroline)manganese(II) 4-hydroxybenzoate monohydrate (Su *et al.*, 2005) and in aqua(3-hydroxybenzoato)bis(1,10-phenanthroline)manganese(II) 3-hydroxybenzoate (Pan *et al.*, 2006). Within the molecule two phen ligands are nearly perpendicular to each other, the dihedral angle being 78.68 (18)°. The uncoordinated HBA anion links with the complex cation *via* O—H···O hydrogen bonding (Table 2).

π-π stacking is observed in the crystal structure (Fig. 2 and 3). The face-to-face separation between parallel N1-phen and N1^A^-phen rings is 3.454 (19) Å [symmetry code: (A) 1 - *x*,-*y*,-*z*]; face-to-face separation between parallel N3-phen and N3^B^-phen rings is 3.435 (7) Å [symmetry code: (B) 2 - *x*,1 - *y*,1 - *z*]. The extensive hydrogen bonding network (Table 2) helps to stabilize the crystal structure.

### Experimental

Co(CH_3_COO)_2_^.^4H_2_O (0.24 g, 1 mmol), sodium 3-hydroxybenzoate (0.16 g, 1 mmol) and phen (0.20 g, 1 mmol) was dissolved in water (15 ml). The solution was refluxed for 5 h, and filtered after cooling to room temperature. Single crystals of the title compound were obtained after 5 d.

### Refinement

The coordinated HBA anion is disordered over two sites, with the hydroxyl group located on the opposite position; occupancies were initially refined and converged to 0.588:0.412, and were fixed as 0.6 and 0.4, respectively, in final cycles of refinement. Accordingly, lattice water molecule O5W is disordered over two sites. One disordered component O5WA is hydrogen bonded to the O3A while the other component O5WB is hydrogen bonded to the other lattice water molecules. Occupancies for O5WA and O5WB were set as 0.6 and 0.4, respectively. The *C*—O_carboxyl_ distances in the disordered HBA components were restrained as 1.25±0.01 Å. The thermal parameters for C1A, C1B, C7A and C7B were constrained to be the same. H atoms of water molecules were placed in chemically sensible positions on the basis of hydrogen bonding interactions, *U*_iso_(H) = 1.5*U*_eq_(O). Aromatic H atoms were placed in calculated positions with C—H = 0.93 Å and refined in riding mode with *U*_iso_(H) = 1.2*U*_eq_(C).

### Crystal data

**Table:**

[Co(C_7_H_5_O_3_)(C_12_H_8_N_2_)_2_(H_2_O)](C_7_H_5_O_3_)·5H_2_O	*Z* = 2
*M**_r_* = 801.65	*F*_000_ = 834
Triclinic, *P*1	*D*_x_ = 1.448 Mg m^−^^3^
Hall symbol: -P 1	Mo *K*α radiation λ = 0.71073 Å
*a* = 12.3404 (12) Å	Cell parameters from 6882 reflections
*b* = 12.6844 (16) Å	θ = 2.5–25.0º
*c* = 13.561 (2) Å	µ = 0.54 mm^−^^1^
α = 101.507 (5)º	*T* = 295 (2) K
β = 101.781 (4)º	Prism, pink
γ = 111.823 (7)º	0.32 × 0.28 × 0.20 mm
*V* = 1838.4 (4) Å^3^	

### Data collection

**Table:**

Rigaku R-AXIS RAPID IP diffractometer	6450 independent reflections
Radiation source: fine-focus sealed tube	4935 reflections with *I* > 2σ(*I*)
Monochromator: graphite	*R*_int_ = 0.050
Detector resolution: 10.00 pixels mm^-1^	θ_max_ = 25.2º
*T* = 295(2) K	θ_min_ = 1.6º
ω scans	*h* = −14→14
Absorption correction: multi-scan(ABSCOR; Higashi, 1995)	*k* = −15→15
*T*_min_ = 0.850, *T*_max_ = 0.905	*l* = −16→16
11865 measured reflections	

### Refinement

**Table:**

Refinement on *F*^2^	Secondary atom site location: difference Fourier map
Least-squares matrix: full	Hydrogen site location: inferred from neighbouring sites
*R*[*F*^2^ > 2σ(*F*^2^)] = 0.070	H-atom parameters constrained
*wR*(*F*^2^) = 0.247	*w* = 1/[σ^2^(*F*_o_^2^) + (0.196*P*)^2^] where *P* = (*F*_o_^2^ + 2*F*_c_^2^)/3
*S* = 1.02	(Δ/σ)_max_ = 0.001
6450 reflections	Δρ_max_ = 0.42 e Å^−^^3^
577 parameters	Δρ_min_ = −0.69 e Å^−^^3^
4 restraints	Extinction correction: none
Primary atom site location: structure-invariant direct methods	

### Special details

**Table:**

Geometry. All e.s.d.'s (except the e.s.d. in the dihedral angle between two l.s. planes) are estimated using the full covariance matrix. The cell e.s.d.'s are taken into account individually in the estimation of e.s.d.'s in distances, angles and torsion angles; correlations between e.s.d.'s in cell parameters are only used when they are defined by crystal symmetry. An approximate (isotropic) treatment of cell e.s.d.'s is used for estimating e.s.d.'s involving l.s. planes.
Refinement. Refinement of *F*^2^ against ALL reflections. The weighted *R*-factor *wR* and goodness of fit *S* are based on *F*^2^, conventional *R*-factors *R* are based on *F*, with *F* set to zero for negative *F*^2^. The threshold expression of *F*^2^ > σ(*F*^2^) is used only for calculating *R*-factors(gt) *etc*. and is not relevant to the choice of reflections for refinement. *R*-factors based on *F*^2^ are statistically about twice as large as those based on *F*, and *R*- factors based on ALL data will be even larger.

### Fractional atomic coordinates and isotropic or equivalent isotropic displacement parameters (Å^2^)

**Table:**

	*x*	*y*	*z*	*U*_iso_*/*U*_eq_	Occ. (<1)
Co	0.60509 (5)	0.22032 (5)	0.34437 (4)	0.0424 (2)	
N1	0.6556 (3)	0.4006 (3)	0.4352 (3)	0.0471 (8)	
N2	0.7823 (3)	0.2680 (3)	0.4520 (3)	0.0483 (8)	
N3	0.6048 (3)	0.0665 (3)	0.2420 (3)	0.0465 (8)	
N4	0.6569 (3)	0.2830 (3)	0.2146 (3)	0.0456 (8)	
O1	0.5335 (3)	0.1227 (3)	0.4432 (2)	0.0551 (8)	
H1A	0.5394	0.1655	0.5102	0.083*	
H1B	0.4574	0.0855	0.4018	0.083*	
O11	0.5066 (4)	0.2421 (4)	0.6154 (3)	0.0735 (10)	
O12	0.5309 (4)	0.4010 (4)	0.7405 (3)	0.0783 (11)	
O13	0.8806 (3)	0.1749 (4)	0.7731 (3)	0.0789 (11)	
H13	0.8129	0.0979	0.7327	0.118*	
C1A	0.2218 (10)	0.1511 (11)	0.1942 (9)	0.0508 (15)	0.60
C2A	0.2545 (14)	0.2317 (14)	0.1350 (17)	0.059 (4)	0.60
H2A	0.3368	0.2788	0.1455	0.071*	0.60
C3A	0.1661 (14)	0.2409 (12)	0.0626 (12)	0.075 (4)	0.60
C4A	0.0412 (15)	0.1750 (12)	0.0547 (10)	0.078 (3)	0.60
H4A	−0.0191	0.1842	0.0084	0.094*	0.60
C5A	0.0067 (13)	0.0990 (14)	0.1122 (14)	0.071 (4)	0.60
H5A	−0.0756	0.0550	0.1044	0.085*	0.60
C6A	0.0984 (13)	0.0895 (12)	0.1828 (10)	0.067 (3)	0.60
H6A	0.0763	0.0399	0.2243	0.081*	0.60
C7A	0.3203 (11)	0.1353 (11)	0.2694 (14)	0.0508 (15)	0.60
O1A	0.4263 (12)	0.1942 (12)	0.2653 (15)	0.053 (4)	0.60
O2A	0.2963 (17)	0.0622 (16)	0.3208 (15)	0.071 (4)	0.60
O3A	0.1860 (7)	0.3081 (6)	−0.0005 (5)	0.0820 (19)	0.60
H1	0.2626	0.3600	0.0150	0.123*	0.60
C1B	0.2094 (15)	0.1263 (15)	0.2168 (11)	0.0508 (15)	0.40
C2B	0.0995 (14)	0.0641 (15)	0.2263 (16)	0.060 (4)	0.40
H2B	0.0939	0.0132	0.2678	0.072*	0.40
C3B	−0.0032 (12)	0.0716 (15)	0.1789 (19)	0.084 (5)	0.40
C4B	0.006 (2)	0.147 (2)	0.112 (3)	0.083 (9)	0.40
H4B	−0.0614	0.1600	0.0832	0.100*	0.40
C5B	0.120 (2)	0.204 (2)	0.0892 (18)	0.088 (8)	0.40
H5B	0.1265	0.2467	0.0403	0.106*	0.40
C6B	0.218 (2)	0.192 (2)	0.144 (2)	0.058 (6)	0.40
H6B	0.2929	0.2276	0.1330	0.070*	0.40
C7B	0.3307 (16)	0.1191 (18)	0.268 (2)	0.0508 (15)	0.40
O1B	0.4321 (19)	0.202 (2)	0.278 (2)	0.070 (8)	0.40
O2B	0.311 (2)	0.037 (2)	0.310 (2)	0.059 (5)	0.40
O3B	−0.1123 (10)	0.0191 (15)	0.2019 (15)	0.142 (6)	0.40
H2	−0.1000	0.0015	0.2657	0.213*	0.40
C11	0.5705 (5)	0.3309 (5)	0.6973 (4)	0.0636 (13)	
C12	0.6997 (5)	0.3480 (4)	0.7466 (3)	0.0565 (11)	
C13	0.7313 (4)	0.2527 (5)	0.7315 (4)	0.0576 (12)	
H14	0.6737	0.1784	0.6861	0.069*	
C14	0.8459 (5)	0.2668 (5)	0.7826 (4)	0.0609 (12)	
C15	0.9346 (5)	0.3798 (5)	0.8489 (4)	0.0678 (14)	
H15	1.0126	0.3905	0.8844	0.081*	
C16	0.9037 (5)	0.4740 (6)	0.8601 (4)	0.0753 (16)	
H16	0.9632	0.5494	0.9014	0.090*	
C17	0.7878 (5)	0.4609 (5)	0.8125 (4)	0.0655 (13)	
H17	0.7683	0.5255	0.8239	0.079*	
C21	0.5936 (4)	0.4671 (5)	0.4228 (4)	0.0560 (11)	
H21	0.5167	0.4318	0.3724	0.067*	
C22	0.6393 (5)	0.5870 (5)	0.4822 (5)	0.0688 (14)	
H22	0.5941	0.6304	0.4708	0.083*	
C23	0.7511 (5)	0.6384 (5)	0.5569 (4)	0.0669 (13)	
H23	0.7827	0.7176	0.5976	0.080*	
C24	0.8187 (4)	0.5724 (4)	0.5726 (3)	0.0540 (11)	
C25	0.9376 (5)	0.6207 (4)	0.6496 (4)	0.0633 (13)	
H25	0.9728	0.6994	0.6923	0.076*	
C26	0.9991 (5)	0.5519 (5)	0.6602 (4)	0.0623 (13)	
H26	1.0754	0.5846	0.7111	0.075*	
C27	0.9495 (4)	0.4305 (5)	0.5953 (3)	0.0553 (11)	
C28	1.0094 (5)	0.3559 (5)	0.6036 (4)	0.0659 (14)	
H28	1.0854	0.3847	0.6540	0.079*	
C29	0.9566 (5)	0.2421 (5)	0.5383 (4)	0.0681 (14)	
H29	0.9958	0.1926	0.5442	0.082*	
C30	0.8434 (4)	0.2004 (5)	0.4624 (4)	0.0589 (12)	
H30	0.8087	0.1229	0.4172	0.071*	
C31	0.8347 (4)	0.3812 (4)	0.5182 (3)	0.0471 (10)	
C32	0.7674 (4)	0.4524 (4)	0.5088 (3)	0.0462 (10)	
C33	0.5792 (4)	−0.0416 (4)	0.2556 (4)	0.0555 (11)	
H33	0.5602	−0.0538	0.3166	0.067*	
C34	0.5797 (5)	−0.1353 (5)	0.1835 (5)	0.0694 (14)	
H34	0.5600	−0.2085	0.1963	0.083*	
C35	0.6090 (5)	−0.1212 (5)	0.0941 (4)	0.0690 (14)	
H35	0.6101	−0.1838	0.0459	0.083*	
C36	0.6376 (4)	−0.0094 (5)	0.0760 (4)	0.0595 (12)	
C37	0.6691 (5)	0.0159 (6)	−0.0152 (4)	0.0748 (16)	
H37	0.6707	−0.0438	−0.0665	0.090*	
C38	0.6962 (5)	0.1239 (6)	−0.0284 (4)	0.0705 (15)	
H38	0.7169	0.1372	−0.0885	0.085*	
C39	0.6943 (4)	0.2189 (5)	0.0470 (4)	0.0567 (12)	
C40	0.7194 (5)	0.3336 (5)	0.0369 (4)	0.0646 (13)	
H40	0.7421	0.3523	−0.0209	0.077*	
C41	0.7105 (5)	0.4165 (5)	0.1111 (4)	0.0696 (15)	
H41	0.7251	0.4913	0.1032	0.084*	
C42	0.6791 (4)	0.3893 (4)	0.2002 (4)	0.0584 (12)	
H42	0.6736	0.4472	0.2505	0.070*	
C43	0.6633 (3)	0.1978 (4)	0.1388 (3)	0.0461 (10)	
C44	0.6349 (4)	0.0821 (4)	0.1533 (3)	0.0455 (9)	
O1W	0.5758 (4)	0.5521 (5)	0.9411 (4)	0.1013 (15)	
H1AW	0.5657	0.5095	0.8695	0.152*	
H1BW	0.5728	0.4970	0.9730	0.152*	
O2W	0.2485 (9)	0.1055 (6)	0.5219 (5)	0.172 (3)	
H2AW	0.3251	0.1504	0.5602	0.258*	
H2BW	0.2501	0.0760	0.4575	0.258*	
O3W	0.2752 (6)	0.3142 (7)	0.7040 (6)	0.154 (3)	
H3AW	0.3587	0.3523	0.7218	0.231*	
H3BW	0.2548	0.3157	0.7643	0.231*	
O4W	0.0758 (10)	0.0332 (12)	0.6051 (12)	0.280 (6)	
H4AW	0.1166	0.1149	0.6377	0.419*	
H4BW	0.1266	0.0219	0.5707	0.419*	
O5WA	0.1205 (9)	0.1918 (12)	−0.2128 (10)	0.148 (4)	0.60
H5A1	0.0408	0.1483	−0.2283	0.222*	0.60
H5A2	0.1433	0.2299	−0.1480	0.222*	0.60
O5WB	0.198 (2)	0.2812 (15)	0.8858 (16)	0.170 (8)	0.40
H5B1	0.1772	0.3385	0.9099	0.255*	0.40
H5B2	0.2755	0.3110	0.9254	0.255*	0.40

### Atomic displacement parameters (Å^2^)

**Table:**

	*U*^11^	*U*^22^	*U*^33^	*U*^12^	*U*^13^	*U*^23^
Co	0.0402 (4)	0.0439 (4)	0.0377 (3)	0.0111 (3)	0.0110 (2)	0.0161 (2)
N1	0.0447 (19)	0.053 (2)	0.0433 (18)	0.0178 (17)	0.0141 (15)	0.0188 (16)
N2	0.0487 (19)	0.048 (2)	0.0475 (19)	0.0182 (17)	0.0114 (16)	0.0201 (16)
N3	0.0439 (18)	0.049 (2)	0.0393 (17)	0.0112 (16)	0.0127 (15)	0.0161 (15)
N4	0.0440 (18)	0.0445 (19)	0.0447 (18)	0.0128 (15)	0.0128 (15)	0.0192 (15)
O1	0.0611 (18)	0.0548 (18)	0.0506 (16)	0.0186 (15)	0.0242 (14)	0.0235 (14)
O11	0.080 (2)	0.088 (3)	0.0471 (18)	0.034 (2)	0.0138 (17)	0.0177 (18)
O12	0.090 (3)	0.083 (3)	0.068 (2)	0.047 (2)	0.025 (2)	0.016 (2)
O13	0.059 (2)	0.079 (3)	0.093 (3)	0.0218 (19)	0.0203 (19)	0.032 (2)
C1A	0.040 (2)	0.059 (3)	0.042 (2)	0.016 (2)	0.0118 (19)	0.000 (2)
C2A	0.049 (8)	0.057 (9)	0.061 (6)	0.019 (6)	0.008 (6)	0.012 (6)
C3A	0.075 (9)	0.051 (6)	0.075 (7)	0.012 (6)	0.009 (6)	0.014 (5)
C4A	0.072 (8)	0.077 (8)	0.082 (8)	0.037 (6)	0.014 (7)	0.018 (6)
C5A	0.050 (6)	0.051 (9)	0.092 (9)	0.009 (6)	0.008 (5)	0.018 (8)
C6A	0.061 (7)	0.053 (7)	0.083 (8)	0.019 (5)	0.016 (6)	0.025 (5)
C7A	0.040 (2)	0.059 (3)	0.042 (2)	0.016 (2)	0.0118 (19)	0.000 (2)
O1A	0.040 (7)	0.059 (7)	0.055 (7)	0.025 (6)	0.005 (4)	0.011 (4)
O2A	0.051 (7)	0.086 (10)	0.058 (6)	0.002 (6)	0.024 (4)	0.031 (7)
O3A	0.089 (5)	0.073 (4)	0.065 (4)	0.023 (4)	−0.003 (3)	0.030 (3)
C1B	0.040 (2)	0.059 (3)	0.042 (2)	0.016 (2)	0.0118 (19)	0.000 (2)
C2B	0.027 (6)	0.039 (8)	0.102 (14)	0.012 (5)	0.010 (8)	0.012 (8)
C3B	0.033 (7)	0.068 (10)	0.141 (17)	0.018 (6)	0.011 (8)	0.034 (11)
C4B	0.065 (11)	0.047 (14)	0.125 (19)	0.036 (12)	0.005 (11)	0.003 (15)
C5B	0.055 (13)	0.091 (17)	0.084 (14)	0.043 (12)	−0.035 (12)	−0.010 (11)
C6B	0.052 (13)	0.068 (15)	0.044 (9)	0.031 (10)	0.001 (9)	−0.002 (11)
C7B	0.040 (2)	0.059 (3)	0.042 (2)	0.016 (2)	0.0118 (19)	0.000 (2)
O1B	0.042 (10)	0.099 (16)	0.046 (8)	−0.002 (8)	0.010 (7)	0.041 (10)
O2B	0.034 (6)	0.067 (9)	0.058 (8)	0.016 (5)	0.010 (5)	0.000 (6)
O3B	0.053 (6)	0.160 (13)	0.241 (19)	0.037 (7)	0.063 (9)	0.114 (13)
C11	0.077 (3)	0.066 (3)	0.045 (2)	0.022 (3)	0.021 (2)	0.027 (2)
C12	0.068 (3)	0.059 (3)	0.043 (2)	0.021 (2)	0.021 (2)	0.024 (2)
C13	0.055 (3)	0.065 (3)	0.044 (2)	0.014 (2)	0.016 (2)	0.023 (2)
C14	0.062 (3)	0.066 (3)	0.057 (3)	0.023 (3)	0.022 (2)	0.029 (2)
C15	0.054 (3)	0.071 (3)	0.054 (3)	0.004 (3)	0.011 (2)	0.020 (2)
C16	0.077 (4)	0.070 (4)	0.056 (3)	0.007 (3)	0.020 (3)	0.017 (3)
C17	0.075 (3)	0.063 (3)	0.056 (3)	0.020 (3)	0.027 (3)	0.024 (2)
C21	0.059 (3)	0.064 (3)	0.053 (2)	0.032 (2)	0.021 (2)	0.021 (2)
C22	0.080 (4)	0.069 (3)	0.077 (3)	0.044 (3)	0.032 (3)	0.031 (3)
C23	0.078 (3)	0.055 (3)	0.068 (3)	0.026 (3)	0.029 (3)	0.015 (2)
C24	0.055 (3)	0.053 (3)	0.046 (2)	0.013 (2)	0.020 (2)	0.014 (2)
C25	0.068 (3)	0.045 (3)	0.048 (2)	0.006 (2)	0.008 (2)	0.002 (2)
C26	0.056 (3)	0.057 (3)	0.047 (2)	0.004 (2)	0.004 (2)	0.014 (2)
C27	0.047 (2)	0.066 (3)	0.045 (2)	0.011 (2)	0.0112 (19)	0.029 (2)
C28	0.052 (3)	0.078 (4)	0.057 (3)	0.017 (3)	0.006 (2)	0.029 (3)
C29	0.062 (3)	0.075 (4)	0.071 (3)	0.033 (3)	0.010 (3)	0.034 (3)
C30	0.055 (3)	0.053 (3)	0.066 (3)	0.022 (2)	0.012 (2)	0.023 (2)
C31	0.044 (2)	0.051 (2)	0.041 (2)	0.0097 (19)	0.0135 (17)	0.0233 (19)
C32	0.051 (2)	0.051 (2)	0.0370 (19)	0.016 (2)	0.0169 (18)	0.0204 (18)
C33	0.059 (3)	0.058 (3)	0.055 (2)	0.022 (2)	0.023 (2)	0.026 (2)
C34	0.077 (3)	0.053 (3)	0.079 (3)	0.024 (3)	0.028 (3)	0.024 (3)
C35	0.080 (4)	0.061 (3)	0.070 (3)	0.034 (3)	0.029 (3)	0.014 (3)
C36	0.055 (3)	0.068 (3)	0.055 (3)	0.026 (2)	0.020 (2)	0.015 (2)
C37	0.088 (4)	0.090 (4)	0.056 (3)	0.043 (3)	0.038 (3)	0.019 (3)
C38	0.071 (3)	0.091 (4)	0.051 (3)	0.028 (3)	0.030 (2)	0.023 (3)
C39	0.051 (2)	0.074 (3)	0.047 (2)	0.021 (2)	0.021 (2)	0.028 (2)
C40	0.061 (3)	0.081 (4)	0.057 (3)	0.024 (3)	0.024 (2)	0.040 (3)
C41	0.057 (3)	0.068 (3)	0.078 (4)	0.012 (3)	0.013 (3)	0.047 (3)
C42	0.051 (3)	0.056 (3)	0.060 (3)	0.010 (2)	0.012 (2)	0.030 (2)
C43	0.0334 (19)	0.058 (3)	0.041 (2)	0.0113 (18)	0.0091 (16)	0.0220 (19)
C44	0.038 (2)	0.053 (2)	0.043 (2)	0.0153 (19)	0.0111 (17)	0.0191 (19)
O1W	0.104 (3)	0.128 (4)	0.080 (3)	0.045 (3)	0.037 (2)	0.047 (3)
O2W	0.281 (10)	0.121 (5)	0.108 (4)	0.075 (6)	0.063 (5)	0.039 (4)
O3W	0.123 (5)	0.190 (7)	0.217 (7)	0.092 (5)	0.082 (5)	0.122 (6)
O4W	0.204 (10)	0.316 (15)	0.370 (16)	0.166 (11)	0.087 (10)	0.102 (13)
O5WA	0.097 (7)	0.185 (11)	0.149 (9)	0.044 (7)	0.049 (6)	0.044 (9)
O5WB	0.23 (2)	0.096 (11)	0.167 (16)	0.043 (13)	0.094 (16)	0.019 (11)

### Geometric parameters (Å, °)

**Table:**

Co—O1	2.107 (3)	C15—C16	1.374 (9)
Co—O1A	2.116 (14)	C15—H15	0.9300
Co—O1B	2.05 (2)	C16—C17	1.375 (8)
Co—N1	2.154 (4)	C16—H16	0.9300
Co—N2	2.153 (3)	C17—H17	0.9300
Co—N3	2.155 (4)	C21—C22	1.404 (7)
Co—N4	2.185 (3)	C21—H21	0.9300
N1—C21	1.345 (6)	C22—C23	1.361 (8)
N1—C32	1.353 (5)	C22—H22	0.9300
N2—C30	1.345 (6)	C23—C24	1.400 (7)
N2—C31	1.359 (6)	C23—H23	0.9300
N3—C33	1.348 (6)	C24—C32	1.415 (6)
N3—C44	1.359 (5)	C24—C25	1.439 (7)
N4—C42	1.336 (6)	C25—C26	1.361 (8)
N4—C43	1.373 (6)	C25—H25	0.9300
O1—H1A	0.9347	C26—C27	1.435 (7)
O1—H1B	0.8775	C26—H26	0.9300
O11—C11	1.268 (6)	C27—C31	1.402 (6)
O12—C11	1.266 (7)	C27—C28	1.408 (8)
O13—C14	1.377 (7)	C28—C29	1.359 (8)
O13—H13	0.9714	C28—H28	0.9300
C1A—C6A	1.388 (18)	C29—C30	1.392 (7)
C1A—C2A	1.415 (18)	C29—H29	0.9300
C1A—C7A	1.522 (17)	C30—H30	0.9300
C2A—C3A	1.37 (3)	C31—C32	1.443 (6)
C2A—H2A	0.9300	C33—C34	1.384 (7)
C3A—O3A	1.319 (14)	C33—H33	0.9300
C3A—C4A	1.422 (19)	C34—C35	1.360 (8)
C4A—C5A	1.36 (2)	C34—H34	0.9300
C4A—H4A	0.9300	C35—C36	1.414 (7)
C5A—C6A	1.38 (2)	C35—H35	0.9300
C5A—H5A	0.9300	C36—C44	1.415 (7)
C6A—H6A	0.9300	C36—C37	1.431 (7)
C7A—O2A	1.253 (8)	C37—C38	1.343 (8)
C7A—O1A	1.261 (8)	C37—H37	0.9300
O3A—H1	0.8786	C38—C39	1.428 (8)
C1B—C2B	1.34 (2)	C38—H38	0.9300
C1B—C6B	1.40 (3)	C39—C40	1.411 (8)
C1B—C7B	1.56 (2)	C39—C43	1.421 (6)
C2B—C3B	1.35 (2)	C40—C41	1.356 (8)
C2B—H2B	0.9300	C40—H40	0.9300
C3B—O3B	1.39 (2)	C41—C42	1.410 (7)
C3B—C4B	1.44 (4)	C41—H41	0.9300
C4B—C5B	1.45 (4)	C42—H42	0.9300
C4B—H4B	0.9300	C43—C44	1.441 (6)
C5B—C6B	1.36 (3)	O1W—H1AW	0.9705
C5B—H5B	0.9300	O1W—H1BW	0.8857
C6B—H6B	0.9300	O2W—H2AW	0.8762
C7B—O2B	1.253 (10)	O2W—H2BW	0.8867
C7B—O1B	1.259 (10)	O3W—H3AW	0.9143
O3B—H2	0.9326	O3W—H3BW	0.9019
C11—C12	1.512 (7)	O4W—H4AW	0.9284
C12—C13	1.393 (7)	O4W—H4BW	0.8901
C12—C17	1.402 (7)	O5WA—H5A1	0.8846
C13—C14	1.369 (7)	O5WA—H5A2	0.8442
C13—H14	0.9300	O5WB—H5B1	0.8813
C14—C15	1.407 (7)	O5WB—H5B2	0.8883
			
O1B—Co—O1	87.8 (6)	C12—C13—H14	119.5
O1B—Co—O1A	4.0 (12)	C13—C14—O13	123.4 (5)
O1—Co—O1A	89.6 (4)	C13—C14—C15	119.8 (5)
O1B—Co—N2	164.3 (8)	O13—C14—C15	116.9 (5)
O1—Co—N2	85.65 (13)	C16—C15—C14	118.6 (5)
O1A—Co—N2	168.3 (5)	C16—C15—H15	120.7
O1B—Co—N1	89.9 (8)	C14—C15—H15	120.7
O1—Co—N1	102.07 (13)	C15—C16—C17	122.4 (5)
O1A—Co—N1	93.0 (4)	C15—C16—H16	118.8
N2—Co—N1	77.61 (13)	C17—C16—H16	118.8
O1B—Co—N3	101.1 (8)	C16—C17—C12	118.6 (5)
O1—Co—N3	91.94 (13)	C16—C17—H17	120.7
O1A—Co—N3	97.5 (4)	C12—C17—H17	120.7
N2—Co—N3	93.34 (14)	N1—C21—C22	123.1 (4)
N1—Co—N3	162.56 (13)	N1—C21—H21	118.4
O1B—Co—N4	88.3 (7)	C22—C21—H21	118.4
O1—Co—N4	167.18 (13)	C23—C22—C21	118.5 (5)
O1A—Co—N4	85.8 (5)	C23—C22—H22	120.7
N2—Co—N4	101.06 (13)	C21—C22—H22	120.7
N1—Co—N4	90.11 (13)	C22—C23—C24	120.2 (5)
N3—Co—N4	76.88 (13)	C22—C23—H23	119.9
C21—N1—C32	118.2 (4)	C24—C23—H23	119.9
C21—N1—Co	127.9 (3)	C23—C24—C32	118.1 (4)
C32—N1—Co	113.8 (3)	C23—C24—C25	123.3 (5)
C30—N2—C31	117.9 (4)	C32—C24—C25	118.6 (5)
C30—N2—Co	128.6 (3)	C26—C25—C24	120.4 (4)
C31—N2—Co	113.4 (3)	C26—C25—H25	119.8
C33—N3—C44	116.8 (4)	C24—C25—H25	119.8
C33—N3—Co	128.1 (3)	C25—C26—C27	121.9 (4)
C44—N3—Co	115.1 (3)	C25—C26—H26	119.0
C42—N4—C43	118.3 (4)	C27—C26—H26	119.0
C42—N4—Co	128.5 (3)	C31—C27—C28	116.9 (5)
C43—N4—Co	113.2 (3)	C31—C27—C26	119.2 (5)
Co—O1—H1A	117.8	C28—C27—C26	124.0 (4)
Co—O1—H1B	96.8	C29—C28—C27	120.3 (5)
H1A—O1—H1B	111.6	C29—C28—H28	119.8
C14—O13—H13	112.7	C27—C28—H28	119.8
C6A—C1A—C2A	118.0 (12)	C28—C29—C30	119.4 (5)
C6A—C1A—C7A	121.5 (11)	C28—C29—H29	120.3
C2A—C1A—C7A	120.4 (12)	C30—C29—H29	120.3
C3A—C2A—C1A	120.6 (12)	N2—C30—C29	122.5 (5)
C3A—C2A—H2A	119.7	N2—C30—H30	118.8
C1A—C2A—H2A	119.7	C29—C30—H30	118.8
O3A—C3A—C2A	125.6 (13)	N2—C31—C27	123.0 (4)
O3A—C3A—C4A	116.0 (13)	N2—C31—C32	117.8 (4)
C2A—C3A—C4A	118.3 (12)	C27—C31—C32	119.2 (4)
C5A—C4A—C3A	122.5 (13)	N1—C32—C24	121.9 (4)
C5A—C4A—H4A	118.8	N1—C32—C31	117.4 (4)
C3A—C4A—H4A	118.8	C24—C32—C31	120.7 (4)
C4A—C5A—C6A	117.6 (13)	N3—C33—C34	123.4 (4)
C4A—C5A—H5A	121.2	N3—C33—H33	118.3
C6A—C5A—H5A	121.2	C34—C33—H33	118.3
C5A—C6A—C1A	122.7 (13)	C35—C34—C33	120.5 (5)
C5A—C6A—H6A	118.7	C35—C34—H34	119.8
C1A—C6A—H6A	118.7	C33—C34—H34	119.8
O2A—C7A—O1A	124.2 (15)	C34—C35—C36	118.7 (5)
O2A—C7A—C1A	123.1 (13)	C34—C35—H35	120.6
O1A—C7A—C1A	112.4 (15)	C36—C35—H35	120.6
C7A—O1A—Co	134.9 (13)	C35—C36—C44	117.5 (4)
C3A—O3A—H1	114.8	C35—C36—C37	123.7 (5)
O5WB^i^—O3A—H1	82.4	C44—C36—C37	118.8 (5)
C2B—C1B—C6B	119.4 (18)	C38—C37—C36	121.4 (5)
C2B—C1B—C7B	125.2 (14)	C38—C37—H37	119.3
C6B—C1B—C7B	114.7 (18)	C36—C37—H37	119.3
C1B—C2B—C3B	123.2 (18)	C37—C38—C39	122.0 (5)
C1B—C2B—H2B	118.4	C37—C38—H38	119.0
C3B—C2B—H2B	118.4	C39—C38—H38	119.0
C2B—C3B—O3B	121.8 (19)	C40—C39—C43	116.6 (5)
C2B—C3B—C4B	117.7 (17)	C40—C39—C38	124.9 (5)
O3B—C3B—C4B	120.1 (17)	C43—C39—C38	118.5 (5)
C3B—C4B—C5B	120.5 (19)	C41—C40—C39	120.4 (4)
C3B—C4B—H4B	119.7	C41—C40—H40	119.8
C5B—C4B—H4B	119.7	C39—C40—H40	119.8
C6B—C5B—C4B	115 (2)	C40—C41—C42	119.9 (5)
C6B—C5B—H5B	122.3	C40—C41—H41	120.0
C4B—C5B—H5B	122.3	C42—C41—H41	120.0
C5B—C6B—C1B	123 (2)	N4—C42—C41	122.1 (5)
C5B—C6B—H6B	118.5	N4—C42—H42	119.0
C1B—C6B—H6B	118.5	C41—C42—H42	119.0
O2B—C7B—O1B	128 (3)	N4—C43—C39	122.7 (4)
O2B—C7B—C1B	111.4 (18)	N4—C43—C44	117.8 (4)
O1B—C7B—C1B	119 (2)	C39—C43—C44	119.5 (4)
C7B—O1B—Co	129 (2)	N3—C44—C36	123.2 (4)
C3B—O3B—H2	112.5	N3—C44—C43	117.0 (4)
O12—C11—O11	124.0 (5)	C36—C44—C43	119.8 (4)
O12—C11—C12	119.7 (5)	H1AW—O1W—H1BW	100.9
O11—C11—C12	116.2 (5)	H2AW—O2W—H2BW	106.1
C13—C12—C17	119.4 (5)	H3AW—O3W—H3BW	107.9
C13—C12—C11	121.2 (4)	H4AW—O4W—H4BW	100.6
C17—C12—C11	119.3 (5)	H5A1—O5WA—H5A2	104.6
C14—C13—C12	121.0 (5)	H5B1—O5WB—H5B2	101.6
C14—C13—H14	119.5		

Symmetry codes: (i) *x*, *y*, *z*−1.

### Hydrogen-bond geometry (Å, °)

**Table:**

*D*—H···*A*	*D*—H	H···*A*	*D*···*A*	*D*—H···*A*
O1—H1A···O11	0.93	1.77	2.671 (5)	162
O1—H1B···O2A	0.88	1.95	2.78 (2)	159
O3A—H1···O1W^ii^	0.88	1.78	2.650 (10)	170
O13—H13···O2A^iii^	0.97	1.86	2.81 (2)	166
O1W—H1AW···O12	0.97	1.85	2.810 (7)	168
O1W—H1BW···O1W^iv^	0.89	2.33	2.833 (8)	116
O2W—H2AW···O11	0.88	1.99	2.851 (11)	167
O2W—H2BW···O2A	0.89	2.04	2.90 (2)	162
O3W—H3AW···O12	0.91	1.92	2.824 (10)	168
O3W—H3BW···O5WA^v^	0.90	1.94	2.589 (16)	127
O3W—H3BW···O5WB	0.90	1.98	2.87 (3)	168
O4W—H4AW···O5WA^v^	0.93	2.05	2.66 (2)	122
O4W—H4AW···O3W	0.93	2.39	3.284 (17)	161
O4W—H4BW···O2W	0.89	1.82	2.569 (18)	140
O5WA—H5A1···O13^vi^	0.88	2.13	2.851 (13)	138
O5WA—H5A2···O3A	0.84	1.91	2.754 (14)	176
O5WB—H5B2···O1W^iv^	0.89	2.16	2.955 (17)	148

Symmetry codes: (ii) −*x*+1, −*y*+1, −*z*+1; (iii) −*x*+1, −*y*, −*z*+1; (iv) −*x*+1, −*y*+1, −*z*+2; (v) *x*, *y*, *z*+1; (vi) *x*−1, *y*, *z*−1.
